# A single column separation method for barium isotope analysis of geologic and hydrologic materials with complex matrices

**DOI:** 10.1186/s12932-021-00077-z

**Published:** 2021-08-11

**Authors:** R. M. Matecha, R. C. Capo, B. W. Stewart, R. L. Thompson, J. A. Hakala

**Affiliations:** 1 Department of Geology & Environmental Science, University of Pittsburgh, Pittsburgh, PA 15260 USA; 2 National Energy Technology Laboratory, 626 Cochrans Mill Road, P.O. Box 10940, Pittsburgh, PA 15236-0940 USA; 3 NETL Support Contractor, 626 Cochrans Mill Road, P.O. Box 10940, Pittsburgh, PA 15236-0940 USA

**Keywords:** Barium, Ba isotopes, Barite, MC-ICPMS, Column chromatography

## Abstract

The increasing significance of barium (Ba) in environmental and geologic research in recent years has led to interest in the application of the Ba isotopic composition as a tracer for natural materials with complex matrices. Most Ba isotope measurement techniques require separation of Ba from the rest of sample prior to analysis. This paper presents a method using readily available materials and disposable columns that effectively separates Ba from a range of geologic and hydrologic materials, including carbonate minerals, silicate rocks, barite, river water, and fluids with high total dissolved solids and organic content such as oil and gas brines, rapidly and without need for an additional cleanup column. The technique involves off-the-shelf columns and cation exchange resin and a two-reagent elution that uses 2.5 N HCl followed by addition of 2.0 N HNO_3_. We present data to show that major matrix elements from almost any natural material are separated from Ba in a single column pass, and that the method also effectively reduces or eliminates isobaric interferences from lanthanum and cerium.

**Supplementary Information:**

The online version contains supplementary material available at 10.1186/s12932-021-00077-z.

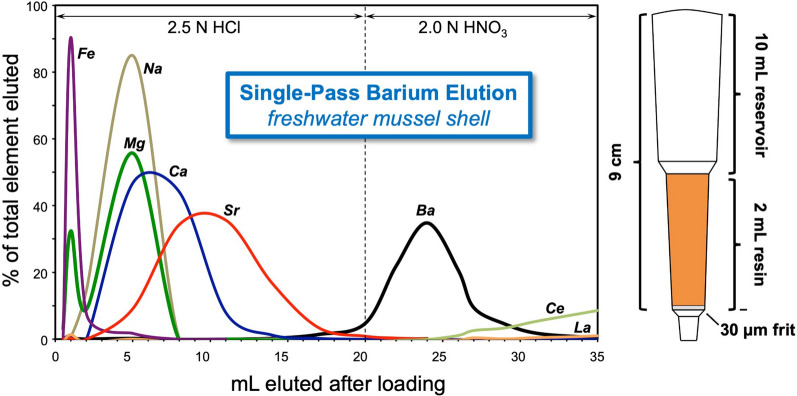

## Introduction

Barium (Ba) is a critical element of interest in studies of oceanic biogeochemical cycling and the diagenetic alteration of sediments (e.g., [1–5]), and the isotopic composition of Ba has been used to understand element cycling in marine, riverine, lacustrine and pedogenic environments [6–16]. More recently, Ba concentrations [17–19] and its isotope signatures [20] have been studied as indicators of Ba sources and mobility related to subsurface water–rock interactions and the fate and transport of contaminants in drilling mud, solid waste and produced waters related to hydraulic fracturing of oil and gas wells.

In order to obtain the Ba isotope composition of a sample via thermal ionization mass spectrometry (TIMS) or multi-collector inductively coupled plasma mass spectrometry (MC-ICPMS), the Ba must be separated from other elements in the sample. High levels of matrix elements will interfere with the ionization and transmission of Ba, and elements with directly overlapping isotopic masses (isobaric interferences, including Xe, La, and Ce) change the measured Ba isotope ratios unless they are removed prior to and/or corrected for during isotope ratio measurement [21–27]. While Xe is only introduced during MC-ICPMS measurement from the atmosphere and Ar gas source, all other interferents (matrix major elements, La, and Ce) must be removed via chemical processing prior to analysis by TIMS or MC-ICPMS. For samples with high ratios of Ba to matrix and/or isobaric interferents (e.g., high-Ba formation waters or samples of barite, BaSO_4_), the chemical separation of Ba is relatively straightforward [6, 14, 20, 24, 28, 29]. However, silicate rock samples can have high rare earth element (REE) to Ba ratios, necessitating robust procedures for removing La and Ce prior to analysis.

Various methods for the separation of Ba for isotopic analysis have been reported in detail. Miyazaki et al. [30] described an elution method using 2.5 N hydrochloric acid (HCl) to separate matrix major elements followed by 1.5 N nitric acid (HNO_3_) to enhance separation of Ba from REE in silicate rock samples. However, two major isotopes of Ba (^136^Ba and ^138^Ba) were excluded from the MC-ICPMS measurement protocol due to interferences from ^138^La, ^136^Ce, and ^138^Ce [30]. Nan et al. [23] used a similar elution method (3.0 N HCl followed by 4.0 N HNO_3_), using two successive columns to fully remove interferents. Elution curves for La and Ce were not reported in this study. For separation of Ba from a range of silicate and carbonate rock types, Nan et al. [31] and Zeng et al. [25] reported using up to three column passes to fully separate Ba from the REE. Tian et al. [28] described a method for separation of Ba from barite using 3 N HCl and 3 N HNO_3_. Because barite consists of ~ 59% Ba by weight, matrix and isobaric interferences are negligible, and the Ba could be purified in a single step.

This paper describes sample preparation and ion exchange chromatography separation of Ba using a two-reagent elution in a single off-the-shelf column that is suitable for the isotopic analysis of Ba in samples with complex matrices and a range of REE/Ba ratios that are encountered in geologic and environmental samples. In addition to barite and seawater, we demonstrate that the method is applicable to low-Ba silicates and carbonate rocks, river water, and high total dissolved solid (TDS) fluids containing organic compounds, such as oil and gas produced waters and other brines. The method uses readily available polypropylene disposable columns and cation exchange resin. We document that this method successfully separates Ba from matrix major elements, as well as La and Ce, in one step, thus necessitating minor to negligible correction for these isobaric interferences during measurement by MC-ICPMS.

## Experimental methods

### Materials and reagents

Experimental work was conducted at the University of Pittsburgh under clean lab conditions. Ultrapure 18.2 MΩ Milli-Q water (MQW) was used for acid dilutions and for washing solids. Teflon beakers and other sample vessels were acid washed, and most sensitive procedures (e.g., sample dissolution, spiking, evaporation, and column separations) were carried out in ULPA-filtered laminar flow hoods. Cation exchange resin (Bio-Rad AG® 50W-X8 200–400 mesh) was pre-cleaned with repeated, sequential applications of MQW, 2% HNO_3_, and 6 N HCl, which were added to the resin in a fluorinated ethylene propylene (FEP) bottle, shaken vigorously, and decanted after settling. Ultrapure (Fisher Optima™) HCl, HNO_3_, hydrofluoric acid (HF) and hydrogen peroxide (H_2_O_2_) were used for sample dissolution and preparation, resin/column preparation, elution and organic matter removal. Ultrapure (J.T. Baker™ Ultrex™) sodium carbonate (Na_2_CO_3_) was used for barite dissolution and seawater Ba precipitation. The Na_2_CO_3_ solution was further purified to remove Ba by using a modification of the method described by Foster et al. [32]; in this method, 1.15 M Na_2_CO_3_ solution is reacted with a 1.1 N calcium chloride solution (CaCl_2_; Thermo Fisher Alfa Aesar™ 99.99% purity), which precipitates Ba along with calcium carbonate, and results in an approximately 1 M Na_2_CO_3_ solution with low Ba levels.

**Fig. 1:**
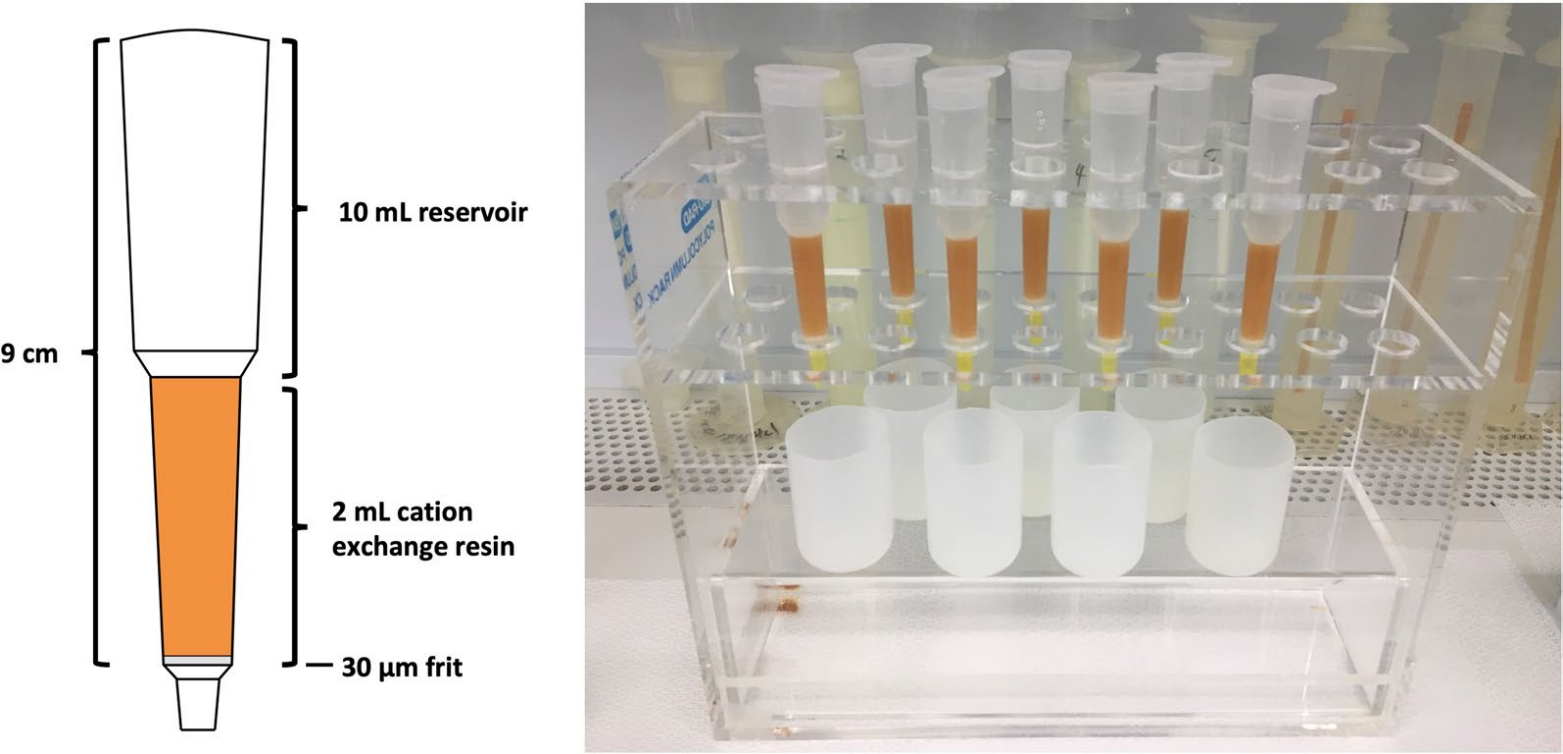
Bio-Rad Poly-Prep® Chromatography Columns used in Ba separation experiments

To prevent sample carryover, we used disposable polypropylene gravity flow ion exchange columns (Bio-Rad Poly-Prep® Chromatography Columns) and disposed of the column and the resin after each use. The columns have a 2 mL bed volume with a 10 mL reservoir and a porous 30 µm polyethylene bed support frit (Fig. 1).

### Samples for Ba separation experiments

Verification of method applicability was conducted using natural materials and standards which reflect a range of matrices encountered in geologic and environmental samples. Silicate rocks (shale, sandstones, igneous rocks) can be problematic for Ba isotopic analysis because of potentially high levels of REE interferents relative to Ba. Carbonate minerals such as limestone and marine shells have high Mg and Ca content, and some freshwater aragonite shells can have high REE relative to Ba [33]. Oil and gas produced waters have high TDS, can contain hydrocarbons and other organic compounds, and can vary greatly in Ba content (0.25 to > 10,000 ppm [34, 35]). For the experiments reported here, fluid samples included seawater (National Research Council Canada seawater reference material NASS-6 collected off the coast of Nova Scotia, Canada), surface water from the Ohio River in Pennsylvania, USA (OR-1204-1a), and produced water from the Marcellus Formation in Greene County, Pennsylvania (M4TFA0518; also used as an internal lab Ba isotope standard). The carbonate sample (PA-LO-102714) was a freshwater mussel shell collected in western Pennsylvania. Silicate rock samples include volcanics from southern Alaska [36], an organic-rich black shale (Marcellus Shale), and USGS standards BCR-2 basalt and AGV-1 andesite [37]. Selected data for these samples are provided in Additional file 1: Table S1.

### Sample dissolution/Ba concentration methods

The carbonate mussel shell was powdered and dissolved in 2.5 N HCl. The silicate rock samples were dissolved under clean lab conditions using HF and HNO_3_ in a microwave digestion system (Milestone Ethos®). Following digestion at 200 °C, the samples were treated with aqua regia and concentrated HNO_3_ to remove fluorides.

Barite was dissolved following a method based on earlier work [14, 28, 38, 39]. In brief, powdered barite was reacted with 1 M purified Na_2_CO_3_ solution (1 mL/10 mg barite) at 95 °C in a sealed Savillex® vial, leading to exchange of SO_4_^2−^ with CO_3_^2−^. After centrifuging, the supernatant was discarded, and the reaction was repeated twice on the remaining residue for a total of 72 h, resulting in a precipitate of barium carbonate (BaCO_3_). The precipitate was rinsed with MQW, evaporated to dryness, and the carbonate dissolved in 3 N HNO_3._ The sample was dried down, redissolved, and diluted to 2% HNO_3_ for elemental analysis.

Because Ba is present at low concentrations (30–175 nM) in seawater [1–3, 7, 9–11], it must be concentrated prior to column separation in order to reduce the matrix load. Barium was extracted from seawater (NASS-6) using a method modified from Foster et al. [32] and others [7, 10–12]. An aliquot of seawater containing ~ 2 µg Ba (~ 200 mL) was spiked with a ^137^Ba–^135^Ba solution (see next section) so that isotope mass fractionation associated with all subsequent processing could be corrected for during measurement. The aliquot was reacted with sufficient 1 M Na_2_CO_3_ solution (purified to remove Ba) to result in precipitation of calcium carbonate, which scavenges Ba from solution. This occurs at a molar ratio of CO_3_^2−^ to Ca^2+^ of approximately 6:1. Once saturation was reached, the reaction occurred nearly instantaneously, and the crystal-fluid mixture was then stirred for 5 min to ensure full removal of Ba. The sample was then decanted into 50 mL centrifuge tubes, rinsing any residue into the tubes using MQW, and centrifuged at 4000 rpm for 5 min. The supernatant was removed by pipette, preserving the precipitate (with most of the Ba) in the centrifuge tube. The precipitate was transferred to a Savillex vial using MQW and a vortex mixer as needed and evaporated to near dryness at 90 °C. The carbonate precipitate was then dissolved in 4 N HCl, evaporated to dryness, and redissolved in 0.5 mL 2.5 N HCl for cation column separation. This method can also be used for separation of Ba from other high TDS fluids and brines with a low Ba concentration and sufficient Ca for precipitation.

### Barium double spike

A calibrated double Ba isotope spike was added to dissolved samples prior to loading of the columns and was used to correct for mass fractionation during MC-ICPMS analysis. Previous work [6–9, 23, 24, 30, 40–43] has shown that this can correct for mass fractionation during chemical processing, thus obviating the need for full (> 99%) recovery of Ba from the column. In addition, use of a double spike reduces the effects of non-isobaric matrix interferents [24]. Based on error analysis using the double spike tool of Rudge et al. [44], the ^137^Ba–^135^Ba isotope pair was chosen because it optimizes the precise determination of ^138^Ba/^134^Ba, whether obtained by direct measurement or calculated based on the measured fractionation in the ^138^Ba/^136^Ba ratio. The double spike was made using ^135^Ba- and ^137^Ba-enriched carbonate salts from the National Isotope Development Center at Oak Ridge National Laboratory and calibrated by running mixtures of double spike and NIST 3104a. Further details of spike calibration can be found in the Supporting Information of Tieman et al. [20].

### Cation column separation procedure

For some samples with very low REE/Ba (carbonate shell PA-LO-102714 and produced water M4TFA0518), a mixed REE standard was added prior to column experiments in order to test the separation of Ba more rigorously from the isobaric interferents La and Ce. In the experiments reported here, approximately 3 mL of cleaned AG50W 200–400 mesh cation resin suspended in MQW was incrementally added by pipette to the acid cleaned columns until the resin bed was just above the base of the 10 mL column reservoir. The resin bed was acidified with 3 mL of HCl at the normality used for that elution experiment, and the resin adjusted to ensure no air bubbles were left and that the top of the resin bed was aligned with the base of the column reservoir. The resin-filled columns were then cleaned with 5 mL of 6.0 N HCl and equilibrated with 4.5 mL of HCl at the appropriate elution normality, added incrementally.

Prior to loading the sample into the columns, residual organics were removed from the sample by adding 1 mL of 50% H_2_O_2_ drop wise, followed by evaporation to dryness. The sample was then redissolved in 0.5 mL of HCl at the appropriate elution normality, allowed to equilibrate for a minimum of 2 h, and placed in an ultrasonic bath for 30 min to ensure that it was dissolved to the fullest extent possible. Samples were centrifuged in 2 mL polypropylene centrifuge tubes at 4000 rpm for 10 min to prevent any insoluble precipitates from being loaded onto the cation exchange resin bed. The supernatant was added to the prepared column using a pipette with a 1 mL polypropylene tip. The sample matrix (excluding Ba and the REE) was eluted by adding HCl of the appropriate normality in successive increments, and the eluent was collected for elemental analysis. This was followed by the addition of either HCl or HNO_3_, depending on the experiment, and these samples (containing primarily Ba and the REE) were collected for elemental analysis.

### Analysis of column calibration cuts

Elemental chemistry of column calibration cuts was determined on a Thermo Element XR® sector field (SF) ICP-MS at the at the National Energy Technology Laboratory (NETL). The SF-ICP-MS sample introduction system used a Glass Expansion Ezylok Micromist nebulizer and Twister spray chamber. The SF-ICP-MS gas flow rates and mass spectrometer voltages were optimized daily in low resolution mode to obtain the maximum signal intensity while minimizing doubly charged and oxide species. Barium, Sr, and REE were analyzed using the low resolution slit (∆m/m = 300), while second row non-metals and Fe were analyzed using the medium resolution slit (∆m/m = 4000).

All samples were spiked with 10 μg/L indium as an internal standard prior to analysis to account for matrix differences. Samples which contained high TDS were minimally diluted with 2% HNO_3_ prior to analysis to minimize matrix effects affecting the ionization in the plasma. While quantitation was not required to generate the elution curves, limits of detection (LOD) were calculated for all analytes as 3 times the standard deviation of the blank from three consecutive blank runs. These detection limits are listed in Table 1. Data from these experiments are reported in Additional file 1: Tables S2–S5.

**Table 1:** Limits of detection (LOD) for all isotopes monitored in this work by SF-ICP-MS

Medium resolution		Low resolution	
Isotope	LOD, µg/L	Isotope	LOD, µg/L
^23^Na	0.487	^88^Sr	0.0004
^24^Mg	0.341	^137^Ba	0.0069
^44^Ca	0.434	^139^La	0.0001
^56^Fe	0.0086	^140^Ce	0.0002

### Ba isotope mass spectrometry method

A subset of samples was analyzed for Ba isotopic composition on a Thermo Neptune Plus® MC-ICPMS at the University of Pittsburgh, part of a joint NETL-University of Pittsburgh facility. For these samples, the separated Ba cut was evaporated to dryness at 90 °C, dissolved in 1 mL of concentrated HNO_3_, and sonicated for 10 min before again being evaporated to dryness. The sample was redissolved in 2% HNO_3_ (2 mL per µg of Ba), sonicated in the sealed container for 60 min, and transferred to an acid cleaned 15 mL centrifuge tube for MC-ICPMS analysis.

We report the isotope ratio of Ba as δ^138^Ba, which is the permil deviation of the ^138^Ba/^134^Ba ratio of a sample from that of NIST Standard Reference Material 3104a:

$${\delta }^{138}Ba={10}^{3}\left[\frac{{\left({}^{138}Ba/{}^{134}Ba\right)}_{sample}}{{\left({}^{138}Ba/{}^{134}Ba\right)}_{3104a}}-1\right]$$

Samples were introduced into the MC-ICPMS using an ESI Apex® desolvating nebulizer. Barium isotopes (^134^Ba, ^135^Ba, ^136^Ba, ^137^Ba, ^138^Ba) were measured simultaneously on five Faraday cups using 10^11^ Ω resistors. Isobaric interferences from Xe, La and Ce were monitored by measurement of ^131^Xe, ^139^La and ^140^Ce on three additional Faraday cups. Xenon, which is present in the atmosphere and can have variable concentrations in the argon gas used in MC-ICP-MS analysis, has interfering masses at 134 and 136 (10.44% and 8.87% of total Xe). Cerium is an isobaric interferent of Ba at mass 136 (^136^Ce = 0.185% of total Ce), and both Ce and La are isobaric interferents at mass 138 (^138^Ce = 0.251% of total Ce and ^138^La = 0.09% of total La). Typical ^138^Ba intensities were 30–40 V for the Apex® desolvating nebulizer (~ 200 V/ppm Ba). No apparent systematic variation in δ^138^Ba was observed within this range of signal intensity. Mass fractionation of the sample was corrected for by iterative normalization to the ^137^Ba/^135^Ba of the double spike using an exponential law [45]. The detector configuration and additional details of the mass spectrometric methods can be found in Supporting Information in Tieman et al. [20].

## Results and discussion

### Optimal separation of Ba

**Fig. 2:**
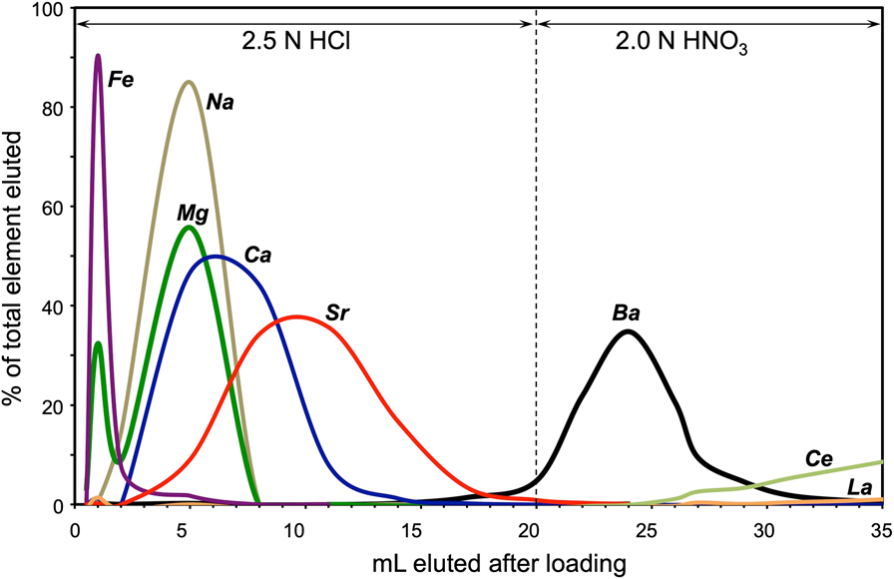
Column matrix separation for major elements and Ba for a 60 mg CaCO_3_ (freshwater mussel shell) sample. Cerium and La were not fully recovered even after 60 mL, so their % elution values are likely overestimated

Combinations of different normalities of HCl (1.5–2.5 N) and HNO_3_ (1–3 N) were tested to optimize the purity of the Ba cut while minimizing the volume and concentration of reagent necessary to elute. Results of selected experiments are reported in Additional file 1: Tables S2–S5; Figures S1, S2. Use of 2.5 N HCl up to the beginning of Ba elution resulted in the optimal separation of major matrix elements from Ba (Fig. 2). Experiments in which only HCl was used for elution (including of Ba) resulted in either overly large volumes of acid being required to fully capture the Ba (e.g*.*, 2.0 N HCl; Additional file 1: Figure S1a) or significant overlap of Ba with the isobaric interferents La and Ce (e.g*.*, 2.5 N HCl; Additional file 1: Figure S1b). van Zuilen et al. [24] report using 2.0 N or 6.4 N HCl to elute Ba, but do not report elution curves for La or Ce. The absence of these peaks during the TIMS measurement [24] may reflect the lower ionization efficiency of these elements relative to Ba rather than separation from the Ba cut. Previous work demonstrated that a better separation of Ba from the REE could be achieved by eluting with HNO_3_ [23, 28, 30]; therefore, we tested different normalities of HNO_3_ for optimal elution of Ba while minimizing overlap of La and Ce. Use of higher normality acid results in a tighter Ba elution peak but greater overlap of REE with Ba (e.g., Additional file 1: Figure S2a,b). The optimal concentration of HNO_3_ to elute Ba from the columns used in these experiments was 2.0 N (Fig. 2).

**Fig. 3:**
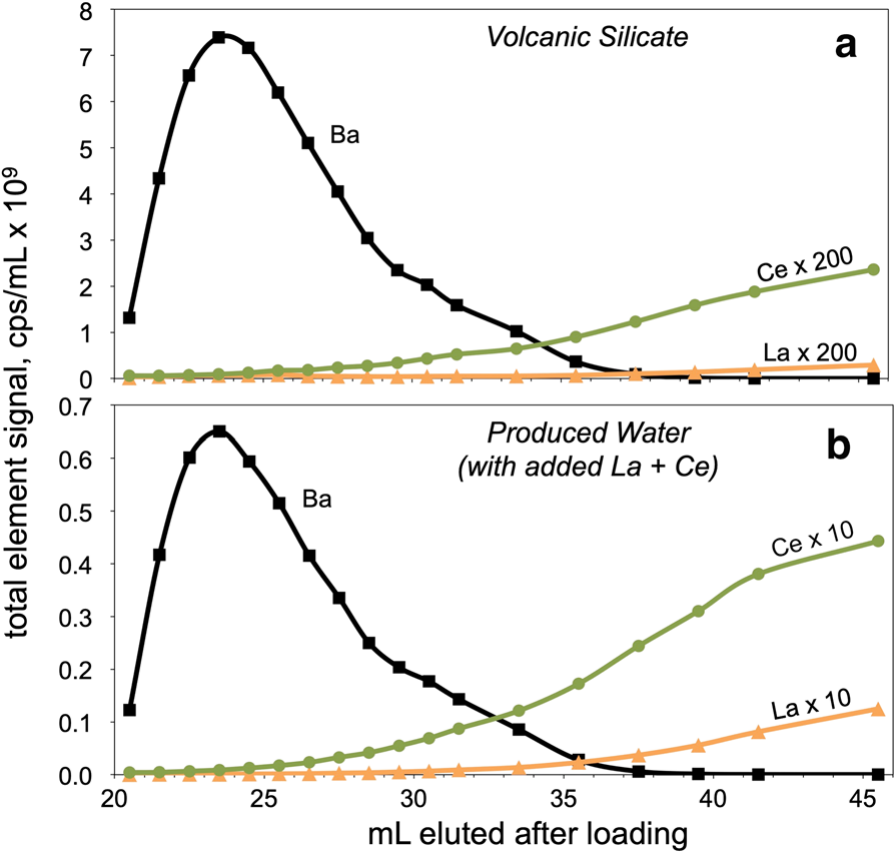
Separation of Ba from isobaric interferents La and Ce during elution of 2 N HNO_3_. The y-axis is the signal intensity (in counts per second by SF-ICP-MS) per mL of each column cut, recalculated to reflect the total element signal rather than just the measured isotope. The La and Ce signals are multiplied by the factors shown on the curves; if plotted at their actual intensities, the curves would be barely distinguishable above baseline. The La/Ba and Ce/Ba of the volcanic silicate sample (**a**) prior to loading was ~ 0.014 and ~ 0.033, respectively, while La and Ce were added to the produced water sample (**b**) prior to loading to generate high La/Ba and Ce/Ba ratios of ~ 0.67

The separation of Ba from REE isobaric interferents was further tested by analyzing Ba elution curves in detail using a dissolved volcanic rock with La/Ba of ~ 0.015 and Ce/Ba of ~ 0.033, and a Marcellus Shale produced water sample in which La and Ce were added to increase La/Ba and Ce/Ba to ~ 0.67 (Fig. 3). Based solely on the measured signal intensities (reported in counts per second) by SF-ICP-MS and the abundance of the measured isotope, the overlap of La with the Ba cut is 0.007% for the volcanic sample and 0.1% for the La-enriched produced water sample. Similarly, the overlap of Ce with the Ba cut is 0.03% and 1.1% for the volcanic sample and Ce-enriched produced water, respectively. In the latter case, such an overlap in Ce would necessitate a correction of ~ 0.3‰ to the δ^138^Ba (for the measurement method described here), which can be done accurately. However, most geologic materials have Ce/Ba ratios (Table 2) considerably lower than the artificially Ce-enriched produced water reported here (Ce/Ba ≈ 0.25). Unusual natural samples with very high Ce/Ba could be run through the same column twice to remove any excess Ce.

**Table 2:** La/Ba and Ce/Ba ranges for various geologic and hydrologic materials

Material	La/Ba	Ce/Ba	References
Upper Continental Crust	0.056	0.12	[47]
Lower Continental Crust	0.071	0.15	[47]
Ocean Ridge Basalts	0.3–53	0.045–10	[48]
Peridotite	0.019–0.48	0.0033–2.0	[49]
Shale	0.023–0.071	0.11–0.15	[47, 50]
Marine carbonate	0.00037–0.077	0.00029–0.20	[51, 52]
Barite	0.000012–0.00055	0.000006–0.00053	[53]
Seawater (average)	0.00037	0.000046	[54]
River water (global average)	0.0052	0.011	[55]

In the optimal separation procedure, a total of 20 mL 2.5 N HCl is used to elute the major matrix elements. Barium is eluted using 12 mL of 2.0 N HNO_3_ (Fig. 2). Major elements were effectively removed prior to the Ba cut; the results were similar for all samples tested (silicate, barite, and fluid samples). The REE isobaric interferents (La and Ce) only begin to elute from the columns after collection of the Ba cut. Additional Ce and La was added to the dissolved aliquot of carbonate (PA-LO-102714) prior to loading in the column to verify separation of Ba from these mass interferents (Fig. 2). Because HNO_3_ so effectively delays release of La and Ce, these elements were not fully removed from the columns even after adding 40 mL of 2 N HNO_3_ (following 20 mL of 2.5 N HCl). For all samples, including biogenic carbonate, silicate volcanic rock, organic-rich shales, and high TDS oil and gas produced brine, use of the method resulted in the release of major elements (e.g., Ca, Na, K, Al) from the sample matrix during the HCl elution. Barium was eluted during the nitric acid step, and La and Ce were effectively held on the resin until the bulk of the Ba was eluted. Using gravity flow, the total time from sample loading to removal of the Ba cut is 3–4 h. With a 24-sample column holder, processing 48 samples/day is easily achievable by a single user. The rate-limiting step is preparing the samples for column work (dissolution, evaporation, precipitation of Ba when needed).

The maximum procedural blank for Ba, determined by the limits of detection by SF-ICP-MS, was 2 ng of Ba, leading to a maximum δ^138^Ba uncertainty of ± 0.002‰ for a 2 µg sample. This assumes the maximum isotopic difference between the sample and blank of 2‰, equivalent to the entire range of δ^138^Ba values measured to date [20]. This is well within the in-run uncertainty of ± 0.03‰ or better for the MC-ICPMS analysis.

### Verification from Ba isotope analysis

Barium isotope analysis of separated samples further demonstrates effective separation. Two silicate rock standards (BCR-2 basalt and AGV-1 andesite) and an internal produced water standard (M4TFA0518) were analyzed after separations using both our previous method [20] and the new method described here. With the previous method (smaller Teflon columns eluted with 2.0 N HCl, with silicates put through a second time to remove REE interferents), we obtained good agreement of the measured δ^138^Ba with published standard values for silicates [20]. The results of the current analyses are shown in Table 3. Because the isobaric interferences from ^136^Ce, ^138^Ce, and ^138^La are monitored continuously during the run using ^139^La and ^140^Ce, we can calculate the total correction to the δ^138^Ba from these interferents. As shown in Table 3, the maximum correction required for Ce is 0.002‰, which is an order of magnitude smaller than the typical in-run uncertainty. The correction required for La is three orders of magnitude smaller than the typical in-run uncertainty. The ratio of total La to total Ba transmitted into the MC-ICPMS after column separation was ~ 3 × 10^–4^ of the pre-column ratio, while the Ce/Ba ratio after column separation was 9–14 × 10^–4^ of the pre-column value. Given that the interfering isotopes only make up 0.09% and 0.49% of the interferent masses measured at ^139^La and ^140^Ce, respectively, this amounts to a negligible or reliably correctable signal.

**Table 3:** Interference corrections and δ^138^Ba values for selected standards

Standard	Unseparated sample^a^		Post-separation^b^		‰ correction from^c^		δ^138^Ba
	La_T_/Ba_T_	Ce_T_/Ba_T_	La_T_/Ba_T_	Ce_T_/Ba_T_	La	Ce	
BCR-2	0.037	0.078	0.000013	0.00011	− 0.00002	0.00211	0.080 ± 0.029
			0.000013	0.00011	− 0.00002	0.00209	0.071 ± 0.026
AGV-1	0.032	0.056	0.000011	0.000048	− 0.00001	0.00090	0.067 ± 0.030
M4TFA0518^d^	n/a	n/a	0.000018	0.000034	− 0.00002	0.00064	0.948 ± 0.034
			0.000018	0.000034	− 0.00002	0.00064	0.936 ± 0.037
			0.000018	0.000035	− 0.00002	0.00066	0.902 ± 0.033
SRM 3104a	n/a	n/a	0.0000053	0.0000016	− 0.00001	0.00003	

n/a no data available

^a^Ratio of total La or Ce to Ba in unseparated sample based on values reported by Raczek et al. [37]

^b^Ratio of total La or Ce to Ba in separated Ba cut based on MC-ICPMS measured intensity of ^138^Ba, ^139^La, and ^140^Ce

^c^Total correction applied to δ^138^Ba value (per mil) from measured ^139^La and ^140^Ce during analysis

^d^Internal lab standard—Marcellus Shale gas well produced water

The δ^138^Ba value of USGS standard BCR-2 (Table 3) that was processed using the methods described in this paper agree well with values reported previously by Nan et al. [23, 31], An et al. [46] and Tieman et al. [20]. The AGV-1 δ^138^Ba value obtained here agrees with values reported by Nan et al. [23, 31], van Zuilen et al. [24], An et al. [46] and Tieman et al. [20].

## Conclusions

We describe a method of Ba separation by cation exchange using readily available, disposable columns suitable for varied sample matrices, including silicates, carbonates, seawater, sulfates, brines, and produced waters. Eluting with 2.5 N HCl ensures the removal of major elements while Ba is still in the column, and the subsequent elution with 2.0 N HNO_3_ separates Ba effectively from REE mass interferents. We show that matrix and isobaric interferents can be removed from most sample types in a single step, with no need for an additional cleanup column. In addition, the use of disposable columns prevents possible cross contamination when dealing with complex sample matrices. The moderate column size and elution volumes allow for rapid (3–4 h) simultaneous separation of multiple samples.

## Supplementary Information

**Additional file 1: Table S1.** Information on samples used for column calibration experiments.** Table S2.** Thermo Element® ICP-MS signal intensity (in counts per second) for a column calibration using *2.0 N HCl *for the entire elution of a dissolved silicate volcanic rock.** Table S3.** Thermo Element® ICP-MS signal intensity (in counts per second) for a column calibration using *2.5 N HCl *for the entire elution of a dissolved silicate volcanic rock.** Table S4.** Thermo Element® ICP-MS signal intensities (in counts per second) for a column calibration using 2.5 N HCl for the first 20 mL, and 3.0 N HNO3 for the remaining elution of a produced water sample with 0.5 μg of La and Ce added.** Table S5.** Thermo Element® ICP-MS signal intensities (in counts per second) for a column calibration using 2.5 N HCl for the first 20 mL, and 2.0 N HNO3 for the remaining elution of dissolved freshwater mussel shell calcium carbonate.** Figure S1. **Examples of column matrix separation for major elements, Ba, and isobaric interferents La and Ce for a volcanic silicate sample using the BIO-RAD Poly-Prep® gravity flow ion exchange columns described in the manuscript.** Figure S2. **Examples of column matrix separation of Ba from isobaric interferents La and Ce for a produced water sample (**a**) and calcium carbonate sample (**b**) using the BIO-RAD Poly-Prep® gravity flow ion exchange columns described in the manuscript.

## Data Availability

The dataset supporting the conclusions of this article is included within the article or in Additional file 1.
