# Supplementary material for: A single column separation method for barium isotope analysis of geologic and hydrologic materials with complex matrices
Source: Geochem Trans. 2021 Aug 11;22:4. doi: 10.1186/s12932-021-00077-z (PMC8359043; doi:10.1186/s12932-021-00077-z)
Supplement: Supplementary file 1 — Additional file 1: Table S1. Information on samples used for column calibration experiments. Table S2. Thermo Element® ICP-MS signal intensity (in counts per second) for a column calibration using 2.0 N HCl for the entire elution of a dissolved silicate volcanic rock. Table S3. Thermo Element® ICP-MS signal intensity (in counts per second) for a column calibration using 2.5 N HCl for the entire elution of a dissolved silicate volcanic rock. Table S4. Thermo Element® ICP-MS signal intensities (in counts per second) for a column calibration using 2.5 N HCl for the first 20 mL, and 3.0 N HNO3 for the remaining elution of a produced water sample with 0.5 μg of La and Ce added. Table S5. Thermo Element® ICP-MS signal intensities (in counts per second) for a column calibration using 2.5 N HCl for the first 20 mL, and 2.0 N HNO3 for the remaining elution of dissolved freshwater mussel shell calcium carbonate. Figure S1. Examples of column matrix separation for major elements, Ba, and isobaric interferents La and Ce for a volcanic silicate sample using the BIO-RAD Poly-Prep® gravity flow ion exchange columns described in the manuscript. Figure S2. Examples of column matrix separation of Ba from isobaric interferents La and Ce for a produced water sample (a) and calcium carbonate sample (b) using the BIO-RAD Poly-Prep® gravity flow ion exchange columns described in the manuscript. [file 12932_2021_77_MOESM1_ESM.pdf]

Supplementary Information

Additional file 1

A single column separation method for barium isotope analysis of geologic and hydrologic materials with complex matrices

Matecha, R. M.[a], Capo, R. C.[a], Stewart, B. W.[a], Thompson, R.L.[b,c], Hakala, J. A.[b]

[a] Department of Geology & Environmental Science, University of Pittsburgh, Pittsburgh, PA 15260, USA

[b] National Energy Technology Laboratory, 626 Cochrans Mill Road, P.O. Box 10940, Pittsburgh, PA 15236-0940, USA

[c]NETL Support Contractor, 626 Cochrans Mill Road, P.O. Box 10940, Pittsburgh, PA 15236-0940, USA

Geochemical Transactions

Additional file 1: Table S1. Information on samples used for column calibration experiments.

Sample	Location	Description	Source	Ba
NASS-6	off coast of Nova Scotia, Canada	open seawater	Nat. Res. Council Canada Ref. Material	184 μg/L
OR-1204-1a	Allegheny County, Pennsylvania	river water	Collected by authors from Ohio River	48.3 μg/L
M4TFA0518	Greene County, Pennsylvania	Marcellus Shale produced water	Collected by authors from active well	16, 400 μg/L
PA-LO-102714	Venango County, Pennsylvania	freshwater mussel shell (aragonite)	Collected by authors from Allegheny River	n/a
Volcanic silicate	southern Alaska	rhyolite-andesite composite	See manuscript reference [36].	200-600 mg/kg[a]
Marcellus shale	Morgantown, West Virginia	organic-rich shale from core	MSEEL[b]	1,300 mg/kg
BCR-2	Bridal Veil Flow Quarry, Oregon	basalt, Columbia River Gorge	USGS Reference Material	677 mg/kg[c]
AGV-1	southern Oregon	andesite	USGS Reference Material	1,200 mg/kg[c]

[a]Range of concentrations in samples used in composite

[b]Marcellus Shale Energy and Environment Laboratory: http://www.mseel.org

[c]Data from Raczek et al., manuscript reference [37].

Additional file 1: Table S2. Thermo Element® ICP-MS signal intensity (in counts per second) for a column calibration using *2.0 N HCl* for the entire elution of a dissolved silicate volcanic rock. All column cuts were diluted to 15 mL prior to analysis. The data (recalculated as fraction of total eluted) are plotted in Fig. S1a.

mL added to column	Cumulative volume	Na23(MR)[a] cps[c]	Mg24(MR) cps	Ca46(MR) cps	Fe56(MR) cps	Sr88(LR)[b] cps	Ba137(LR) cps	La139(LR) cps	Ce140(LR) cps
0.5	0.5	1345851	31152	79	14956	21449	1874	951	1865
0.5	1	2143399	1172966	833256	66505	76611	37784	2330	3673
0.5	1.5	3298254	2010265	2703534	4550209	116555	54060	3222	4343
0.5	2	2299369	432109	94416	34331941	22454	6390	664	1326
2	4	102604622	13477017	92887	164870323	34102	8974	803	1326
5	9	29013226	58298212	53751	62935406	86154	8246	506	1103
5	14	1182372	158898	10401	176996	5891739	7061	433	889
5	19	920620	25719	1125	23286	20384998	5873	489	981
5	24	1291038	12356	322	13850	5031319	5094	392	925
5	29	955782	14252	132	22001	1376019	15593	686	1195
5	34	937024	12539	123	70376	266045	138942	811	1429
5	39	935974	19311	161	47358	58117	612423	884	1579
5	44	859736	9399	146	38899	14227	1048728	934	1179
5	49	1111384	15348	83	104323	7614	896031	942	3222
5	54	891796	9413	108	55600	4482	564025	964	12143
5	59	960798	13612	96	161226	5598	288738	1840	51083
5	64	1017963	10699	102	115298	5052	116921	6168	156128
5	69	1097870	16680	118	40191	8972	46852	19459	351238
5	74	978701	12088	104	24152	4446	18894	48853	606227
5	79	962035	10026	103	21188	3818	7770	105846	809252
5	84	998579	11650	86	17391	6120	3551	183511	855501
5	89	963740	10525	121	20352	3181	1757	263467	776097
5	94	1004586	12021	101	46590	4249	1103	312101	596161
5	99	1075873	23521	154	24493	4135	1106	342744	413320
5	104	1314092	19579	159	32156	3626	2694	303603	265305

[a](MR) = medium resolution data acquisition
[b](LR) = low resolution data acquisition
[c]Intensities of all measured elements are adjusted by the addition of In (10 μg/L) as an internal standard.

Additional file 1: Table S3. Thermo Element® ICP-MS signal intensity (in counts per second) for a column calibration using *2.5 N HCl* for the entire elution of a dissolved silicate volcanic rock. All column cuts were diluted to 15 mL prior to analysis. The data (recalculated as fraction of total eluted) are plotted in Fig. S1b.

mL added to column	Cumulative volume	Na23(MR)[a] cps[c]	Mg24(MR) cps	Ca46(MR) cps	Fe56(MR) cps	Sr88(LR)[b] cps	Ba137(LR) cps	La139(LR) cps	Ce140(LR) cps
0.5	0.5	867209	7225	88	81653	6365	695	533	728
0.5	1	1870432	771774	579577	83810	56004	26533	1654	2391
0.5	1.5	2679033	1421703	2380064	12896816	87592	43599	2227	3178
0.5	2	1785031	407321	88847	52180080	18594	5865	475	695
2	4	92642584	43613830	92384	143970784	34823	7773	453	945
5	9	8837988	15475645	24601	3430386	2008859	8863	622	1028
5	14	1637760	204852	8066	530745	59409591	23356	1754	3334
5	19	1216770	10043	219	66994	3645621	5642	433	939
5	24	1220977	9520	106	55951	578035	42805	422	834
5	29	1174250	7216	96	69159	126380	649453	859	5890
5	34	1223211	7504	82	76929	21327	1389533	3534	85139
5	39	1143903	5927	93	557277	5117	747635	27193	461911
5	44	1300410	6818	104	558539	3829	247749	125093	1116573
5	49	1175395	8540	74	67402	3053	76215	329648	1271331
5	54	1182103	7064	70	75023	3220	21524	487679	893200
5	59	1233130	6529	81	109244	3317	6034	466172	472026
5	64	1135639	9212	74	112123	6343	1946	330360	208386
5	69	1200881	7804	56	158991	3823	1053	191848	78792
5	74	1154308	6951	72	163273	3342	611	96409	30308
5	79	1163780	7965	72	65732	4644	556	41041	11818
5	84	1073772	7124	44	74200	3409	497	18198	5025
5	89	1040628	6698	91	80397	3420	470	8257	2605
5	94	1074826	6325	64	53268	3437	422	4201	1329
5	99	1177035	6997	86	57038	3259	467	2046	747
5	104	1226613	8433	63	22646	5890	595	1565	736

[a](MR) = medium resolution data acquisition
[b](LR) = low resolution data acquisition
[c]Intensities of all measured elements are adjusted by the addition of In (10 μg/L) as an internal standard.

Additional file 1: Table S4. Thermo Element® ICP-MS signal intensities (in counts per second) for a column calibration using 2.5 N HCl for the first 20 mL, and 3.0 N HNO_3 for the remaining elution of a produced water sample with 0.5 µg of La and Ce added. All column cuts were diluted to 15 mL prior to analysis. The data are plotted in modified form in Fig. S2a.

mL added to column	Cumulative volume	Acid added	Na23(MR)[a] cps[d]	Mg24(MR) cps	Al27(MR) cps	Si28(MR) cps	S34(MR) cps	K39(HR)[b] cps	Ca46(MR) cps	Fe56(MR) cps	Sr88(LR)[c] cps	Y89(LR) cps	Ba137(LR) cps	La139(LR) cps	Ce140(LR) cps
0.5	0.5	2.5 N HCl	1492043	45945	357877	785913	1850	165388	311	20067	44310	1151	8105	1330	4722
0.5	1	2.5 N HCl	742757	32799	437742	907572	2063	23385	1196	78056	35872	1368	4947	2356	3704
0.5	1.5	2.5 N HCl	767392	87434	461557	980422	3316	50142	357	28563967	43328	1726	9466	2686	3149
0.5	2	2.5 N HCl	13770512	1569543	462240	843766	2266	54457	344	66812001	28528	1530	5685	1397	2669
2	4	2.5 N HCl	18039462798	295885620	374705	762777	2275	708933	315	85008992	32425	1747	10271	1593	3378
4	8	2.5 N HCl	565707847	34066760	453139	836128	2284	102517143	205402	821468	3119922261	253571	25863	3470	6699
4	12	2.5 N HCl	2272217	62679	504953	991926	2488	457974	52380	48840	29337597781	2293405	23161	773896	1232437
4	16	2.5 N HCl	1884345	46761	543271	869805	2510	182274	1322	40682	9435879424	746155	39390	4943	5080
2	18	2.5 N HCl	966444	50935	426542	800985	1749	45632	424	60106	519531445	37794	179017	4534	2944
1	19	2.5 N HCl	1175030	53198	507444	759669	233862	27369	3858	45081	57584124	8109	306351	1609	3249
1	20	2.5 N HCl	1333316	32744	511000	779674	1678	119882	264	24906	37546196	5873	821483	2135	3791
3	23	3.0 N HNO_3	1181782	37651	445704	853311	3360	52326	369	27484	55222856	31038	596356064	222262	67543
3	26	3.0 N HNO_3	2392347	40307	695960	819588	3299	84323	306	38468	1686395	3487	44929506	203934	1384128
1	27	3.0 N HNO_3	1032505	47620	322902	727392	2010	39402	668	14768	86556	1389	100313	269022	1317992
1	28	3.0 N HNO_3	713521	31694	345333	666968	1947	35224	265	9381	68481	1076	34773	513177	1855077
1	29	3.0 N HNO_3	900980	28848	457254	767579	1856	102383	261	68440	52668	842	22673	826053	2135208
1	30	3.0 N HNO_3	1387276	36861	321981	689030	2007	39912	321	30873	49042	813	17216	1232275	2293941
2	32	3.0 N HNO_3	693647	30267	312130	629267	2039	14625	318	16608	48340	667	16694	3500133	3921648
2	34	3.0 N HNO_3	1139737	34266	566392	768164	2335	26796	303	33638	165926	1459	10029	3860203	2416789
6	40	3.0 N HNO_3	6376279	133000	828991	1024284	3161	58544	501	44775	7848544	2402	116114	6334504	1645992

[a](MR) = medium resolution data acquisition
[b](HR) = high resolution data acquisition
[c](LR) = low resolution data acquisition
[d]Intensities of all measured elements are adjusted by the addition of In (10 µg/L) as an internal standard.

Additional file 1: Table S5. Thermo Element® ICP-MS signal intensities (in counts per second) for a column calibration using 2.5 N HCl for the first 20 mL, and 2.0 N HNO_3 for the remaining elution of dissolved freshwater mussel shell calcium carbonate. Approximately 4 µg of Ba and 0.1 µg of La and Ce were added. All column cuts were diluted to 15 mL prior to analysis. The data are plotted in modified form in Fig. S2b.

mL added to column	Cumulative volume	Acid added	Sr88(LR)[a] cps[b]	Y89(LR) cps	Ba137(LR) cps	La139(LR) cps	Ce140(LR) cps
0.5	0.5	2.5 N HCl	22583	1119	9666	1128	2690
0.5	1	2.5 N HCl	173789	13578	27275	15874	30621
1	2	2.5 N HCl	18429	1203	21445	1582	3902
3	5	2.5 N HCl	33707372	216248	149708	265769	420508
3	8	2.5 N HCl	126533559	475247	17819	2748	5545
3	11	2.5 N HCl	131640311	726303	37059	3126	7625
3	14	2.5 N HCl	61936028	935375	163439	4509	12737
3	17	2.5 N HCl	12827786	924775	678477	8892	25433
3	20	2.5 N HCl	3543712	611454	2153771	15431	47373
2	22	2.0 N HNO_3	967084	167621	6504348	16657	47949
2	24	2.0 N HNO_3	432597	69251	10298462	18604	52097
2	26	2.0 N HNO_3	208285	55374	6137866	20990	68167
2	28	2.0 N HNO_3	82843	24408	1413859	18969	43994
2	30	2.0 N HNO_3	215657	40001	1381888	30727	98209
2	32	2.0 N HNO_3	83876	32250	572540	39631	124554
2	34	2.0 N HNO_3	32373	34802	355294	74117	236415
4	38	2.0 N HNO_3	16677	30004	54131	145403	402060
4	42	2.0 N HNO_3	33023	19679	8051	207276	481636
8	50	2.0 N HNO_3	24583	21694	5562	692876	1077373
8	58	2.0 N HNO_3	27713	10352	6171	911024	849891
2	60	2.0 N HNO_3	17696	2390	4012	231068	149825

[a](LR) = low resolution data acquisition

[b]Intensities of all measured elements are adjusted by the addition of In (10 µg/L) as an internal standard.

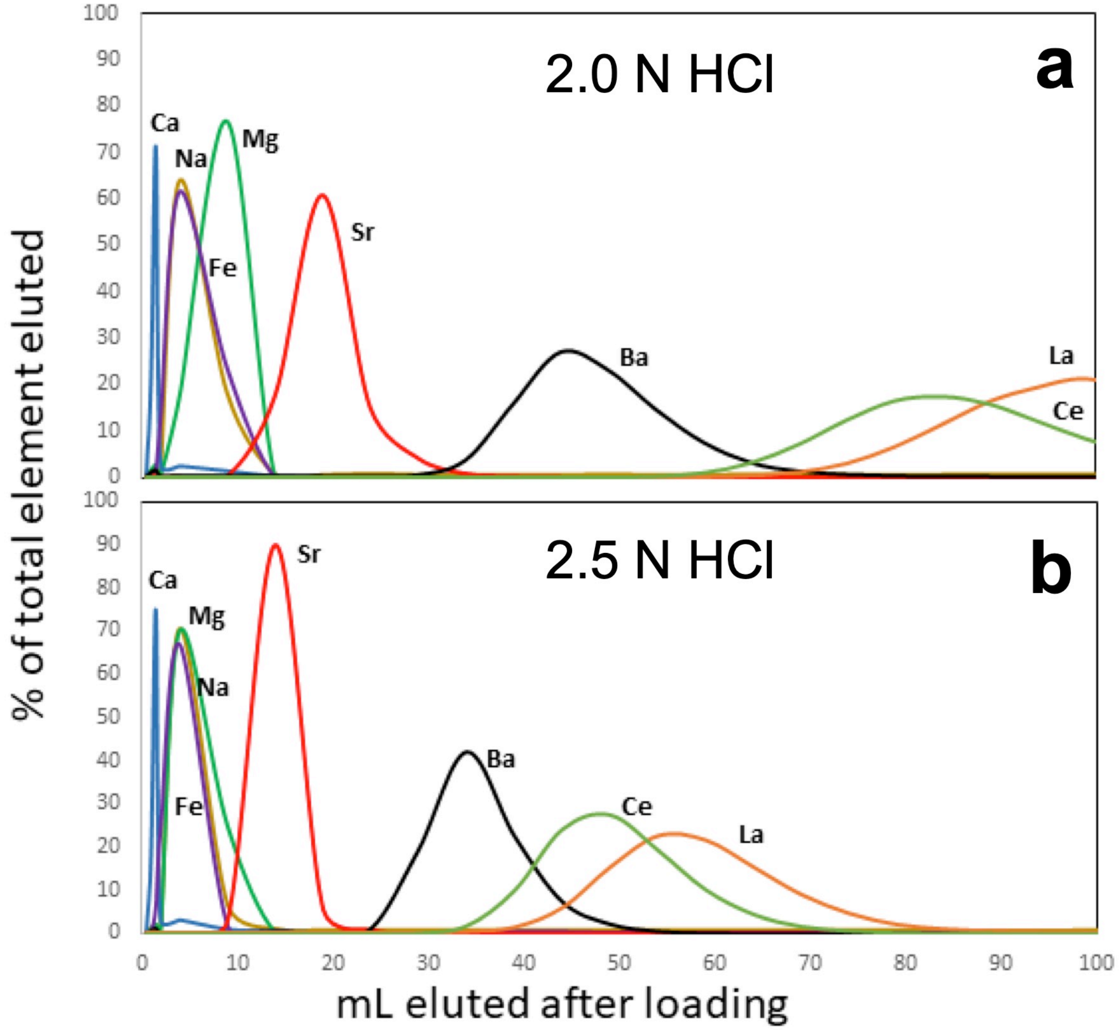

Additional file 1: Fig. S1. Examples of column matrix separation for major elements, Ba, and isobaric interferents La and Ce for a volcanic silicate sample using the BIO-RAD Poly-Prep® gravity flow ion exchange columns described in the manuscript. In both cases, the full elution was carried out with a single normality of hydrochloric acid (HCl). Eluting with 2.0 N HCl (a**)** leads to an effective separation of Ba from both major matrix elements and La and Ce, but requires relatively large volumes of acid. For example, nearly 30 mL of 2.0 N HCl is required to recover the Ba, in a column with a reservoir of only 10 mL. Cerium and La were not fully recovered when using 2.0 N HCl, so their % elution values are estimated. Use of 2.5 N HCl (**b**) leads to a tighter Ba elution curve and effective removal of matrix elements, but a less effective separation of Ba from La and Ce. Raw data used for these plots are provided in Tables S1 and S2.

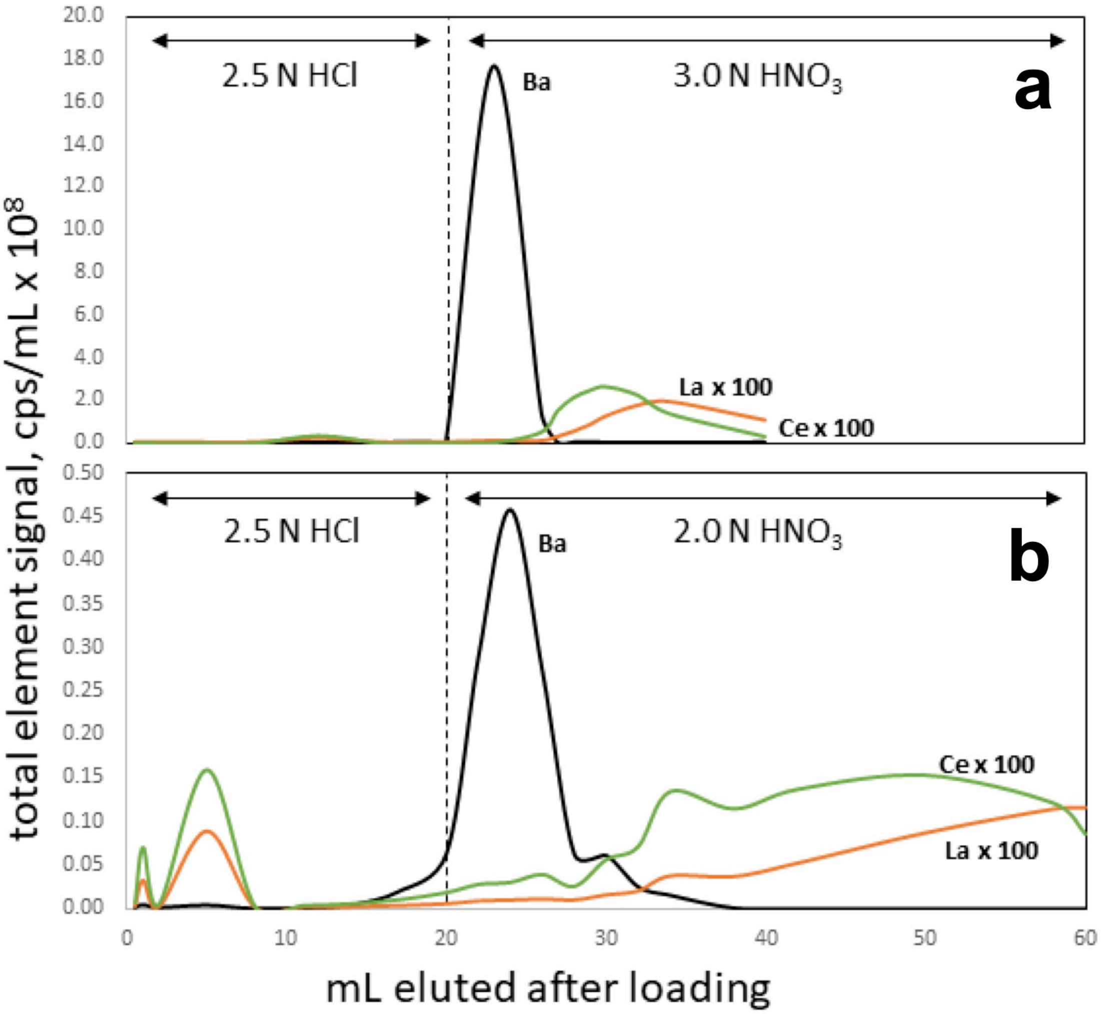

Additional file 1: Fig. S2. Examples of column matrix separation of Ba from isobaric interferents La and Ce for a produced water sample (**a**) and calcium carbonate sample (**b**) using the BIO-RAD Poly-Prep® gravity flow ion exchange columns described in the manuscript. In both cases, major cations (not shown) were eluted with 2.5 N HCl for the first 20 mL; afterward, either 2.0 N or 3.0 N HNO_3 was used to elute the remaining elements. The y-axis is the signal intensity (in counts per second by SF-ICP-MS) per mL of each column cut, recalculated to reflect the total element signature rather than just the measured isotope. Additional La and Ce was added to both samples prior to putting them through the column to provide enough of these elements to evaluate their separation from Ba. The La and Ce signals are multiplied by the factors shown in order to be clearly distinguishable on this diagram. Raw data used for these plots are provided in Tables S3 and S4.
